# Supplementary material for: QTL Mapping and a Transcriptome Integrative Analysis Uncover the Candidate Genes That Control the Cold Tolerance of Maize Introgression Lines at the Seedling Stage
Source: Int J Mol Sci. 2023 Jan 30;24(3):2629. doi: 10.3390/ijms24032629 (PMC9917090; doi:10.3390/ijms24032629)
Supplement: Supplementary file 1 [file ijms-24-02629-s001.zip › Supplementary Figure S1-10.pdf]

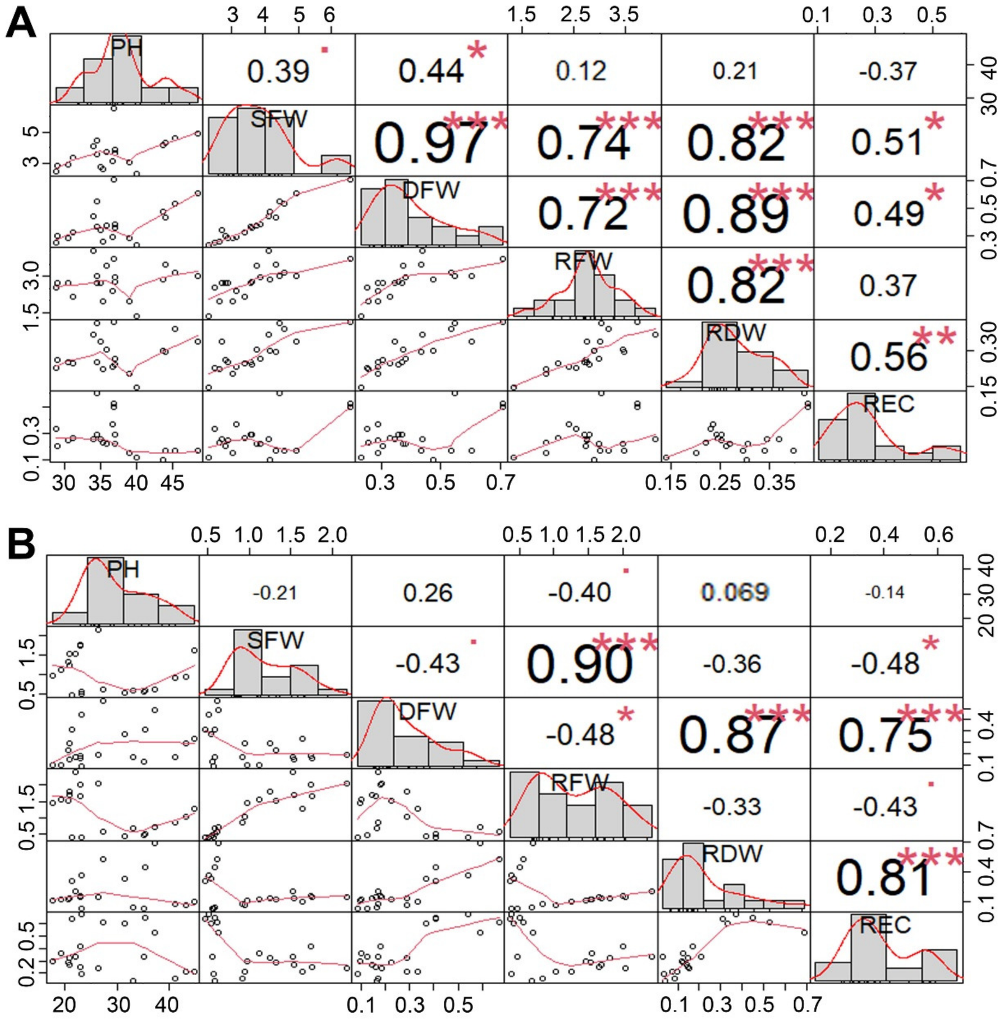


**Supplementary Figure S1**. Population correlation analysis of MTP- maize introgression line.

Note: (A): CK; (B): the low temperature. Plant height/PH, seedling fresh weight/SFW, seedling dry weight/SDW, root fresh weight/RFW, root dry weight/RDW, relative electrical conductivity/REC.

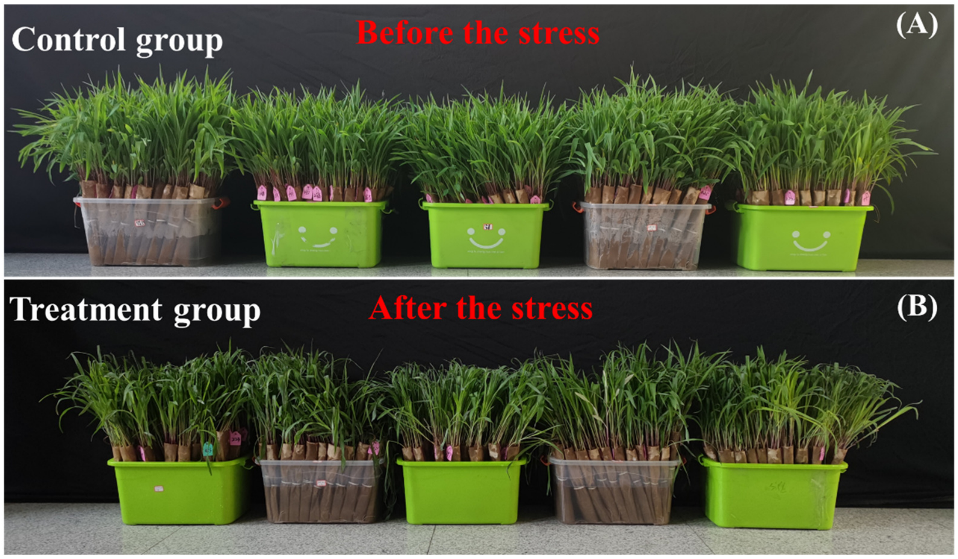


**Supplementary Figure S2**. (A) and (B) showed the plant phenotypes of $F_{2:3}$ populations before treatment and 2℃ for 5 d, respectively.

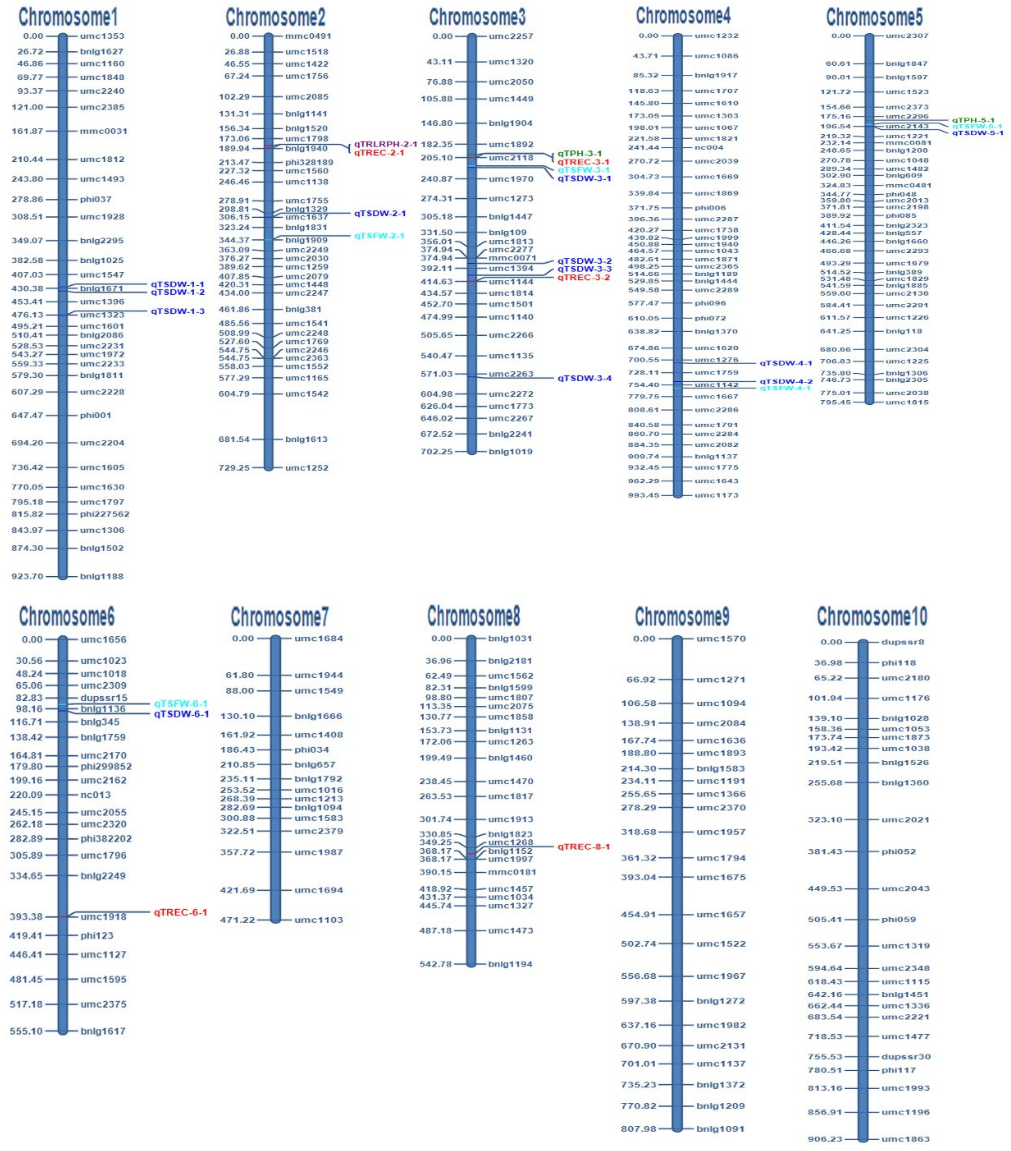


**Supplementary Figure S3**. QTL mapping of seedling-related traits in $F_{2:3}$ population (2℃; 5d)

Note: Plant height/PH, seedling fresh weight/SFW, seedling dry weight/SDW, relative electrical conductivity/REC and root length/RL.

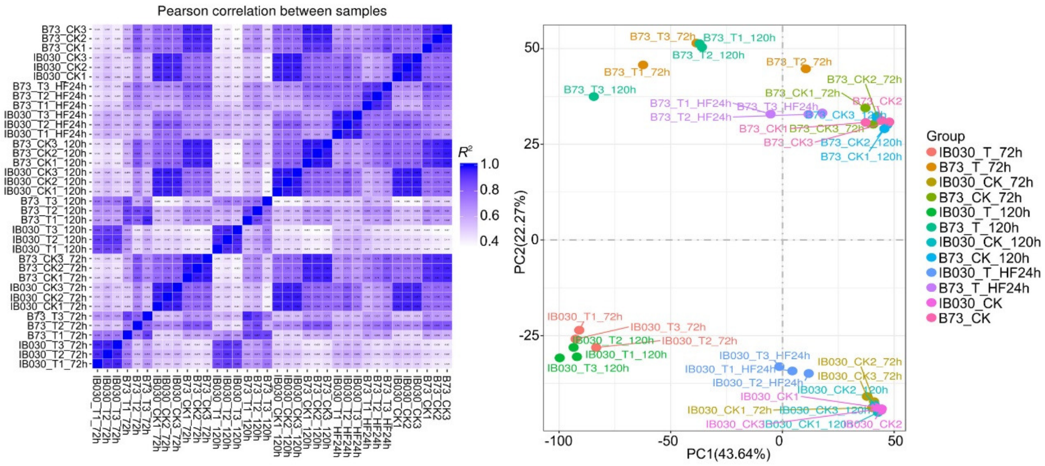


**Supplementary Figure S4**. (A) Heat map of correlation between samples, the horizontal and vertical coordinates in the figure were the square of correlation coefficients of each sample. (B) Diagram of principal component analysis results, the abscissa was the first principal component and the ordinate was the second principal component.

```
B73              ATGGCAACGCCCAAGCTGTCCCCGGTCTCGCCGGTGCGGCAGGACGGCAACGACAAGCCGTGCGCGCCATCCTCCCCCTCCTCCTCCCCGTCGACCGTTC   100
Zm00001d012321   ATGGCAACGCCCAAGCTGTCCCCGGTCTCGCCGGTGCAGCGGGACGGCAACGACAAGCCGTGCGCGCCATCCTCCCCCTCCTCCTCCTCGTCGACCGTTC   100
Consensus        atggcaacgcccaagctgtccccggtctcgccggtgc gc ggacggcaacgacaagccgtgcgcgccatcctccccctcctcctcc cgtcgaccgttc

B73              TGAGTGCGCAGGACGCGTACGAGCAGCACCTGCGCCTGCCGGAGCTGTCGAGCCTGTGGACGGCCGGATGCTTCCCGGAGTGGGCGGGCGAGGGCCTGGT   200
Zm00001d012321   TGAGGGCGCAGGACGCGTACGAGCAGCACCTGCGCCTGCCGGAGCTGTCGAGCCTGTGGACGGCCGGGTGCTTCCCGGAGTGGGCGGGCGAGGGCCTGGT   200
Consensus        tgag gcgcaggacgcgtacgagcagcacctgcgcctgccggagctgtcgagcctgtggacggccgg tgcttcccggagtgggcgggcgagggcctggt

B73              CAAGCCGGCGCTGCAGGCGCTGGAGGTCACCTTCCGCCTCGCGTCCCTGGCGCTCTCCGACCCGCGCGGGCACGCCGGCCGCCGCGAGCTCGCGCGCCGG   300
Zm00001d012321   CAAGCCGGCGCTGCAGGCGCTGGAGGTCACCTTCCGCCTCGCGTCCCTGGCGCTCTCCGACCCGCGCGGGCACGCCGGCCGCCGCGAGCTCGCGCGCCGG   300
Consensus        caagccggcgctgcaggcgctggaggtcaccttccgcctcgcgtccctggcgctctccgacccgcgcgggcacgccggccgccgcgagctcgcgcgccgg

B73              CTGGAGTCCCTCGCGGCGCGGGAGGTGGAGCTGGTGTCGGCGCTCTGCGAGGGCGACGGCCGGGGCGCGCCGCTCGCCGAGCTGAGCGCCTCCGGGGGCG   400
Zm00001d012321   CTGGAGTCCCTCGCGGCGCGGGAGGTGGAGCTGGTGTCGGCGCTCTGCGAGGGCGACGACCGGGGCGCGCCGCTCGCCGAGCTGAGCACCTCGGGGGGCG   400
Consensus        ctggagtccctcgcggcgcgggaggtggagctggtgtcggcgctctgcgagggcgacg ccggggcgcgccgctcgccgagctgagc cctc gggggcg

B73              TGCTCCCGCGGGAGCGCAGCGCGTCCGAGGTGGTGGTGTGGCAGCTGCCCGGGAGCGCCGCCGCGGTCGTGTGCCGGGCCAGCGAGGCCAGCCTGCTCCC   500
Zm00001d012321   TGCTCCCGCGGGAGCGCAGCGCGTCCGAGGTGGTGGTGTGGCAGCTGCCCGGGAGCGCCGCCGCGGTCGTGTGCCGGGCCAGCGAGGCCAGCCTGCTCCC   500
Consensus        tgctcccgcgggagcgcagcgcgtccgaggtggtggtgtggcagctgcccgggagcgccgccgcggtcgtgtgccgggccagcgaggccagcctgctccc

B73              GCGCCTCGCCGCGTGGGAGAAGTCTGAGGCGCTCGCGGCCAGGATCACGTACGCCGTCGAGGGCCAGATGCAGGGCTGCGCCTTCACGCTCGGCCTCGGC   600
Zm00001d012321   GCGCCTCGCCGCGTGGGACAAGTCCGAGGCGCTCGCGGCCAGGATCACGTACGCCGTCGAGGGCCAGATGCAGGGCTGCGCCTTCACGCTCGGCCTCGGC   600
Consensus        gcgcctcgccgcgtggga aagtc gaggcgctcgcggccaggatcacgtacgccgtcgagggccagatgcagggctgcgccttcacgctcggcctcggc

B73              GAGCCCAACCTCGCCGGCAAGCCCGTGCTCGAGTACGACCGCGTCGTGCGCCCGCACGAGCTGCACGCGCTGAAGCCCGACCCTGCGCCGGAGCCCATGT   700
Zm00001d012321   GAGCCCAACCTCGCCGGCAAGCCCGTGCTCGAGTACGACCGCGTCGTGCGCCCGCACGAGCTGCACGCGCTGAAGCCCGAGCCAGCGCCGGAGCCCACGT   700
Consensus        gagcccaacctcgccggcaagcccgtgctcgagtacgaccgcgtcgtgcgcccgcacgagctgcacgcgctgaagcccga cc gcgccggagccca gt

B73              CCGGCTACCGCAACCGGGAGCTCGAGACTCTGTTCACCATGTACCAGATACTCGAGTCCTGGCTCCGCGTCGCGTCGCAGCTGCTCACCCGCCTCGACGA   800
Zm00001d012321   CCGGCTACCGCAACCGGGAGCTCGAGACGCTGTTCACCATGTACCAGATACTCGAGTCCTGGCTGCGCGTCGCGTCGCAGCTGCTCACCCGCCTCGACGA   800
Consensus        ccggctaccgcaaccgggagctcgagac ctgttcaccatgtaccagatactcgagtcctggct cgcgtcgcgtcgcagctgctcacccgcctcgacga

B73              GCGGATCGAAGACAAGTGCTGGGAGGCGGCGGCCGGCGACTGCTGGATCCTGGAGCGCGTGTGGAAGCTGCTCGCGGACGTCGAGGACCTCCACCTGCTG   900
Zm00001d012321   GCGGATCGAAGACAAGTGCTGGGAGGCGGCGGCCGGCGACTGCTGGATCCTGGAGCGCGTGTGGAAGCTGCTCGCGGACGTCGAGGACCTCCACCTGCTG   900
Consensus        gcggatcgaagacaagtgctgggaggcggcggccggcgactgctggatcctggagcgcgtgtggaagctgctcgcggacgtcgaggacctccacctgctg

B73              ATGGACCCGGACGAGTTCCTACGGCTCAAGAGCCAGCTCGCCGTACGAGCGGCGCCGGGGTCTGAGTCCGCGTCCTTCTGTTTCCGGTCCACGGCGCTCC  1000
Zm00001d012321   ATGGACCCGGACGAGTTCCTACGGCTCAAGAGCCAGCTCGCCGTACGAGCGGCGCCTGGGTCTGAGTCCGCGTCCTTCTGTTTCCGGTCCAGGGCGCTCC  1000
Consensus        atggacccggacgagttcctacggctcaagagccagctcgccgtacgagcggcgcc gggtctgagtccgcgtccttctgtttccggtcca ggcgctcc

B73              TGCACGTCGCTAGCGCCACTAGGGACCTCAAGAAGCGTGTGCCCTGGGTGCTCGGTGTCGAGGCGGACCCCAGCGGCGGCCCACGGGTGCAGGAGGCGGC  1100
Zm00001d012321   TGCATGTCGCTAGCGCCACTAGGGACCTCAAGAAGCGTGTGCCCTGGGTGCTCGGTGTCGAGGCGGACCCCAGTGGCGGCCCACGGGTGCAGGAGGCGGC  1100
Consensus        tgca gtcgctagcgccactagggacctcaagaagcgtgtgccctgggtgctcggtgtcgaggcggaccccag ggcggcccacgggtgcaggaggcggc

B73              CATGAAGCTGTACCACAGCCGTAGGCGCGGTGAGGGCGAGGAGGCAGGCAAGGTGGACCTGCTCCAGGCCTTCCAGGCGGTGGAGGTGGCCGTGAGAGCA  1200
Zm00001d012321   CATGAAGCTGTACCACAGCCGTAGGCGCGGTGAGGGCGAGGAGGCAGGCAAGGTGGACCTGCTCCAGGCCTTCCAGGCGGTGGAGGTGGCTGTGAGAGGA  1200
Consensus        catgaagctgtaccacagccgtaggcgcggtgagggcgaggaggcaggcaaggtggacctgctccaggccttccaggcggtggaggtggc gtgagag a

B73              TTCTTCTTCGGGTACCGGCAGCTGGTGGCGGCGGTGATGGGCACGGCGGAGGCGTCGGGCAACCGGGCGCTGTTCGTGCCGGCGGAGGAGATGGATCCGC  1300
Zm00001d012321   TTCTTCTTCGGGTACCGGCAGCTGGTGGCGGCGGTGATGGGCACGGCGGAGGCGTCGGGCAACCGGGCGCTGTTCGTGCCGGCGGAGGAGATGGATCCGC  1300
Consensus        ttcttcttcgggtaccggcagctggtggcggcggtgatgggcacggcggaggcgtcgggcaaccgggcgctgttcgtgccggcggaggagatggatccgc

B73              TCGCCCAAATGTTCCTGGAGCCGCCATACTACCCTAGCCTGGACGCCGCCAAGACGTTTCTAGCGGATTACTGGGTTCAGCTTCAGCAGATGGCGGAGGC  1400
Zm00001d012321   TCGCCCAAATGTTCCTGGAGCCGCCATACTACCCCAGCCTGGACGCCGCCAAGACGTTTCTAGCGGATTACTGGGTTCAGCTTCAGCAGATGGCGGAGGC  1400
Consensus        tcgcccaaatgttcctggagccgccatactaccc agcctggacgccgccaagacgtttctagcggattactgggttcagcttcagcagatggcggaggc

B73              CTCTGCTCCGTCAAGACAAAGCTGA                                                                            1425
Zm00001d012321   CTCTGCTCCGTCAAGACAAAGCTGA                                                                            1425
Consensus        ctctgctccgtcaagacaaagctga
```

**Supplementary Figure S5**. DNA sequence analysis of key gene *Zm00001d012321*

```
Zm00001d012321    MATPKLSPVSPVQRDGNDKPCAPSSPSSSSSTVLRAQDAYEQHLRLPELSSLWTAGCFPEWAGEGLVKPALQALEVTFRLASLALSDPRGHAGRRELARR    100
B73               MATPKLSPVSPVRQDGNDKPCAPSSPSSSPSTVLSAQDAYEQHLRLPELSSLWTAGCFPEWAGEGLVKPALQALEVTFRLASLALSDPRGHAGRRELARR    100
Consensus         matpklspvspv  dgndkpcapsspsss stvl aqdayeqhlrlpelsslwtagcfpewageglvkpalqalevtfrlaslalsdprghagrrelarr

Zm00001d012321    LESLAAREVELVSALCEGDDRGAPLAELSTSGGVLPRERSASEVVVWQLPGSAAAVVCRASEASLLPRLAAWDKSEALAARITYAVEGQMQGCAFTLGLG    200
B73               LESLAAREVELVSALCEGDGRGAPLAELSASGGVLPRERSASEVVVWQLPGSAAAVVCRASEASLLPRLAAWEKSEALAARITYAVEGQMQGCAFTLGLG    200
Consensus         leslaarevelvsalcegd rgaplaels sggvlprersasevvvwqlpgsaaavvcraseasllprlaaw ksealaarityavegqmqgcaftlglg

Zm00001d012321    EPNLAGKPVLEYDRVVRPHELHALKPEPAPEPTSGYRNRELETLFTMYQILESWLRVASQLLTRLDERIEDKCWEAAAGDCWILERVWKLLADVEDLHLL    300
B73               EPNLAGKPVLEYDRVVRPHELHALKPDPAPEPMSGYRNRELETLFTMYQILESWLRVASQLLTRLDERIEDKCWEAAAGDCWILERVWKLLADVEDLHLL    300
Consensus         epnlagkpvleydrvvrphelhalkp papep sgyrnreletlftmyqileswlrvasqlltrlderiedkcweaaagdcwilervwklladvedlhll

Zm00001d012321    MDPDEFLRLKSQLAVRAAPGSESASFCFRSRALLHVASATRDLKKRVPWVLGVEADPSGGPRVQEAAMKLYHSRRRGEGEEAGKVDLLQAFQAVEVAVRG    400
B73               MDPDEFLRLKSQLAVRAAPGSESASFCFRSTALLHVASATRDLKKRVPWVLGVEADPSGGPRVQEAAMKLYHSRRRGEGEEAGKVDLLQAFQAVEVAVRA    400
Consensus         mdpdeflrlksqlavraapgsesasfcfrs allhvasatrdlkkrvpwvlgveadpsggprvqeaamklyhsrrrgegeeagkvdllqafqavevavr

Zm00001d012321    FFFGYRQLVAAVMGTAEASGNRALFVPAEEMDPLAQMFLEPPYYPSLDAAKTFLADYWVQLQQMAEASAPSRQSF                             475
B73               FFFGYRQLVAAVMGTAEASGNRALFVPAEEMDPLAQMFLEPPYYPSLDAAKTFLADYWVQLQQMAEASAPSRQSF                             475
Consensus         fffgyrqlvaavmgtaeasgnralfvpaeemdplaqmfleppyypsldaaktfladywvqlqqmaeasapsrqsf
```

**Supplementary Figure S6**. Amino acid sequence analysis of key gene *Zm00001d012321*

```
B73.txt               ATGGCGTTCCCTCAGATCTCCCGCAGAGCCCTCGGCCTCCTGCTCGTCATCGCCGCAGCGGCCGCGATCGTCTCGCCCGCCACCGCCGACGAGGTGGTGG    100
Zm00001d037590.txt    ATGGCGTTCCCTCAGATCTCCCGCAGAGCCCTCGGCCTCCTGCTCGTCATCGCCGCAGCGGCCGCGATCGTCTCGCCCGCCACCGCCGACGAGGTGGTGG    100
Consensus             atggcgttccctcagatctcccgcagagccctcggcctcctgctcgtcatcgccgcagcggccgcgatcgtctcgcccgccaccgccgacgaggtggtgg

B73.txt               CCCTGACGGAAGCCGACTTCGAGAAGGAGGTCGGCCAGGACCGCGGCGCCCTCGTCGAGTTCTACGCGCCATGGTGTGGCCACTGCAAAAAGCTTGCCCC    200
Zm00001d037590.txt    CCCTGACGGAAGCCGACTTCGAGAAGGAGGTCGGCCAGGACCGCGGCGCCCTCGTCGAGTTCTACGCGCCATGGTGTGGCCACTGCAAAAAGCTTGCCCC    200
Consensus             ccctgacggaagccgacttcgagaaggaggtcggccaggaccgcggcgccctcgtcgagttctacgcgccatggtgtggccactgcaaaaagcttgcccc

B73.txt               CGAGTATGAAAAACTTGGCGCCAGCTTCAAGAAGGCTAAATCTGTACTGATAGCAAAGGTTGACTGTGATGAGCACAAGAGTGTATGCAGCAAGTATGGA    300
Zm00001d037590.txt    CGAGTATGAAAAACTTGGCGCCAGCTTCAAGAAGGCTAAATCTGTACTGATAGCAAAGGTTGACTGTGATGAGCACAAGAGTGTATGCAGCAAGTATGGA    300
Consensus             cgagtatgaaaaacttggcgccagcttcaagaaggctaaatctgtactgatagcaaaggttgactgtgatgagcacaagagtgtatgcagcaagtatgga

B73.txt               GTTTCTGGGTACCCCACAATTCAATGGTTTCCCAAAGGTTCCCTGGAGCCAAAGAAGTATGAGGGTCAACGTTCTGTGGAAGCCCTTGCAGAATTTGTTA    400
Zm00001d037590.txt    GTTTCTGGGTACCCCACAATTCAATGGTTTCCCAAAGGTTCCCTGGAGCCAAAGAAGTATGAGGGTCAACGTTCTGTGGAAGCCCTTGCAGAATTTGTTA    400
Consensus             gtttctgggtaccccacaattcaatggtttcccaaaggttccctggagccaaagaagtatgagggtcaacgttctgtggaagcccttgcagaatttgtta

B73.txt               ACAGCGAAGCAGGTACCAATGTCAAGATAGCTGCCATTCCTTCAAGCGTCGTGGTTCTGACTTCAGAGACCTTTGACTCAATTGTCCTTGATGAAACCAA    500
Zm00001d037590.txt    ACAGCGAAGCAGGTACCAATGTCAAGATAGCTGCCATTCCTTCAAGCGTCGTGGTTCTGACTTCAGAGACCTTTGACTCAATTGTCCTTGATGAAACCAA    500
Consensus             acagcgaagcaggtaccaatgtcaagatagctgccattccttcaagcgtcgtggttctgacttcagagacctttgactcaattgtccttgatgaaaccaa

B73.txt               AGATGTCCTTGTTGAGTTTTATGCCCCATGGTGTGGTCACTGCAAGCATCTTGCTCCGATTTATGAGAAGCTGGCTTCAGTTTTCAAGCAGGACGACGGT    600
Zm00001d037590.txt    AGATGTCCTTGTTGAGTTTTATGCCCCATGGTGTGGTCACTGCAAGCATCTTGCTCCGATTTATGAGAAGCTGGCTTCAGTTTTCAAGCAGGACGACGGT    600
Consensus             agatgtccttgttgagttttatgccccatggtgtggtcactgcaagcatcttgctccgatttatgagaagctggcttcagttttcaagcaggacgacggt

B73.txt               GTTGTGATTGCCAATATTGATGCTGACAAGCACACTGACTTGGCTGAAAAGAATCACAGAACATCTTTAATAATATACCTCTGCAGATATGGTGTTTCTG    700
Zm00001d037590.txt    GTTGTGATTGCCAATATTGATGCTGACAAGCACACTGACTTGGCTGAAAA....................................ATATGGTGTTTCTG    664
Consensus             gttgtgattgccaatattgatgctgacaagcacactgacttggctgaaaa                                    atatggtgtttctg

B73.txt               GTTTCCCTACATTGAAGTTCTTCCCTAAGGGAAACAAAGCCGGTGAAGATTATGATGGTGGCCGGGACTTGGATGACTTTGTCAAGTTCATTAATGAGAA    800
Zm00001d037590.txt    GTTTCCCTACATTGAAGTTCTTCCCTAAGGGAAACAAAGCCGGTGAAGATTATGATGGTGGCCGGGACTTGGATGACTTTGTCAAGTTCATTAATGAGAA    764
Consensus             gtttccctacattgaagttcttccctaagggaaacaaagccggtgaagattatgatggtggccgggacttggatgactttgtcaagttcattaatgagaa

B73.txt               GTGTGGCACCAGCCGGGATCCAAAGGGCCACCTGAATCAAGAGGCTGGGCTTGTGCCAAGCTTGAATCCTCTTGTGAAGGAGTTTCTCAATGCTGCTGAT    900
Zm00001d037590.txt    GTGTGGCACCAGCCGGGATCCAAAGGGCCACCTGAATCAAGAGGCTGGGCTTGTGCCAAGCTTGAATCCTCTTGTGAAGGAGTTTCTCAATGCTGCTGAT    864
Consensus             gtgtggcaccagccgggatccaaagggccacctgaatcaagaggctgggcttgtgccaagcttgaatcctcttgtgaaggagtttctcaatgctgctgat

B73.txt               GACAAGCGGAAGGAAGTCCTCTCTAAAATAGAAGAGGATGTTGCTAAGCTCAGCGGTTCTGCAGCCAAGCATGGAAAGATATATGTGACAGCTGCAAAGA   1000
Zm00001d037590.txt    GACAAGCGGAAGAAAGTCCTCTCTAAAATAGAAGAGGATGTTGCTAAGCTCAGCGGTTCTGCAGCCAAGCATGGAAAGATATATGTGACAGCTGCAAAGA    964
Consensus             gacaagcggaag aagtcctctctaaaatagaagaggatgttgctaagctcagcggttctgcagccaagcatggaaagatatatgtgacagctgcaaaga

B73.txt               AGATCATAGACAAGGGCTCTGACTACACTAAGAAGGAGACTGAGAGGCTTCACCGCATGCTGGAGAAGTCAATCAGTCCTTCCAAAGCTGATGAATTCAT   1100
Zm00001d037590.txt    AGATCATAGACAAGGGCTCTGACTACACTAAGAAGGAGACTGAGAGGCTTCACCGCATGCTGGAGAAGTCAATCAGTCCTTCCAAAGCTGATGAATTCAT   1064
Consensus             agatcatagacaagggctctgactacactaagaaggagactgagaggcttcaccgcatgctggagaagtcaatcagtccttccaaagctgatgaattcat

B73.txt               CGTTAAGAAGAACATTCTTTCGATATTCTCTTCGTGA                                                                1137
Zm00001d037590.txt    CGT.AAGAAGAACATTCTTTCGATATTCTCTTCGTGA                                                                1100
Consensus             cgt aagaagaacattctttcgatattctcttcgtga
```

**Supplementary Figure S7**. DNA sequence analysis of key gene *Zm00001d037590*

```
Zm00001d037590   MAFPQISRRALGLLLVIAAAAAIVSPATADEVVALTEADFEKEVGQDRGALVEFYAPWCGHCKKLAPEYEKLGASFKKAKSVLIAKVDCDEHKSVCSKYG   100
B73              MAFPQISRRALGLLLVIAAAAAIVSPATADEVVALTEADFEKEVGQDRGALVEFYAPWCGHCKKLAPEYEKLGASFKKAKSVLIAKVDCDEHKSVCSKYG   100
Consensus        mafpqisrralglllviaaaaaivspatadevvalteadfekevgqdrgalvefyapwcghckklapeyeklgasfkkaksvliakvdcdehksvcskyg

Zm00001d037590   VSGYPTIQWFPKGSLEPKKYEGQRSVEALAEFVNSEAGTNVKIAAIPSSVVVLTSETFDSIVLDETKDVLVEFYAPWCGHCKHLAPIYEKLASVFKQDDG   200
B73              VSGYPTIQWFPKGSLEPKKYEGQRSVEALAEFVNSEAGTNVKIAAIPSSVVVLTSETFDSIVLDETKDVLVEFYAPWCGHCKHLAPIYEKLASVFKQDDG   200
Consensus        vsgyptiqwfpkgslepkkyegqrsvealaefvnseagtnvkiaaipssvvvltsetfdsivldetkdvlvefyapwcghckhlapiyeklasvfkqddg

Zm00001d037590   VVIANIDADKHTDLAEK............YGVSGFPTLKFFPKGNKAGEDYDGGRDLDDFVKFINEKCGTSRDPKGHLNQEAGLVPSLNPLVKEFLNAAD   288
B73              VVIANIDADKHTDLAEKNHRTSLIIYLCRYGVSGFPTLKFFPKGNKAGEDYDGGRDLDDFVKFINEKCGTSRDPKGHLNQEAGLVPSLNPLVKEFLNAAD   300
Consensus        vvianidadkhtdlaek            ygvsgfptlkffpkgnkagedydggrdlddfvkfinekcgtsrdpkghlnqeaglvpslnplvkeflnaad

Zm00001d037590   DKRKKVLSKIEEDVAKLSGSAAKHGKIYVTAAKKIIDKGSDYTKKETERLHRMLEKSISPSKADEFIVRRTFFRYSLR                         366
B73              DKRKEVLSKIEEDVAKLSGSAAKHGKIYVTAAKKIIDKGSDYTKKETERLHRMLEKSISPSKADEFIVKKNILSIFSS                         378
Consensus        dkrk vlskieedvaklsgsaakhgkiyvtaakkiidkgsdytkketerlhrmleksispskadefiv
```

**Supplementary Figure S8**. Amino acid sequence analysis of key gene *Zm00001d037590*

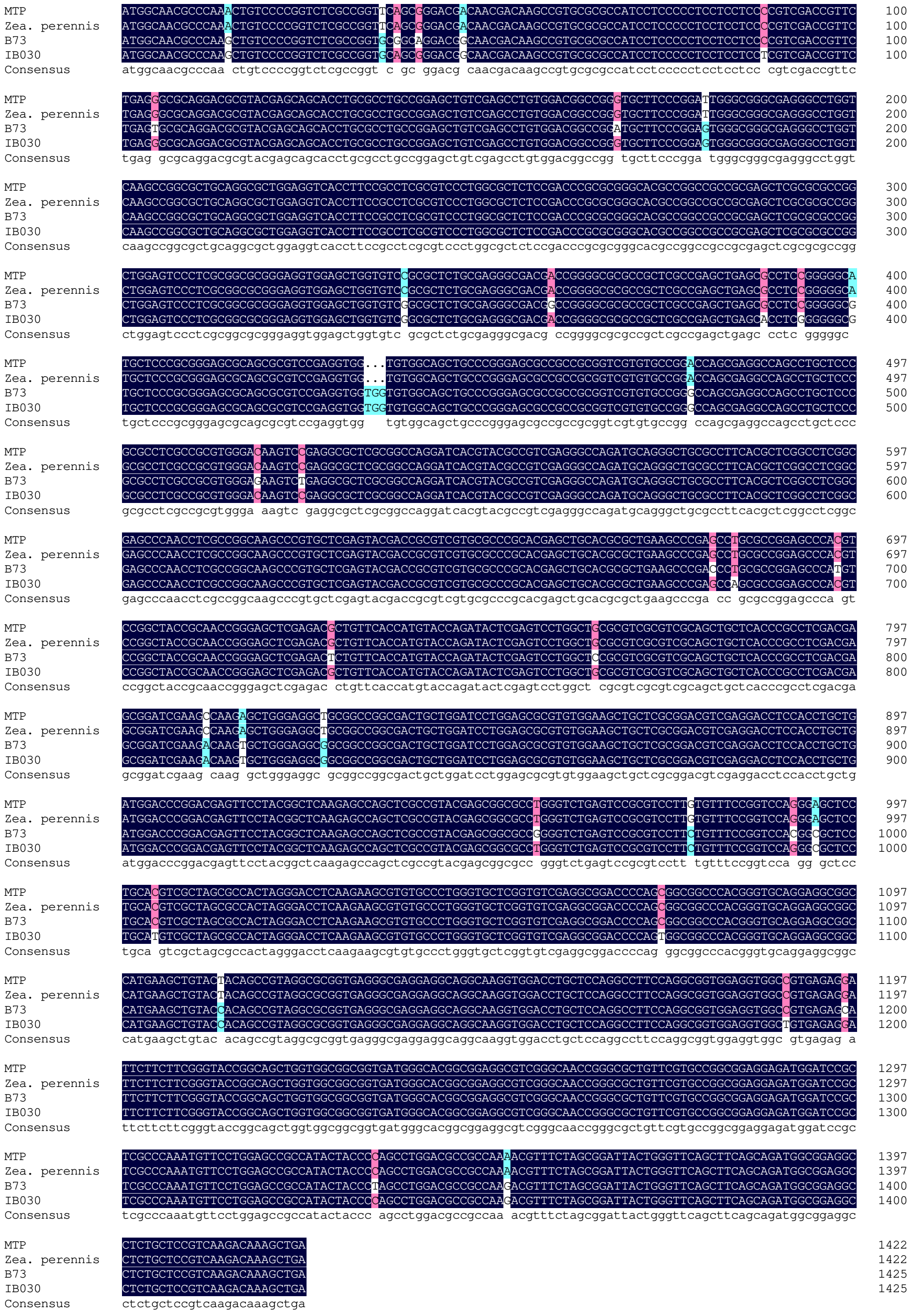

**Supplementary Figure S9**. Sequence comparison of homologous clones of *Zm00001d012321*

Note: MTP (M: maize; T: T. *dactyloides* and P: *Z. perennis*).

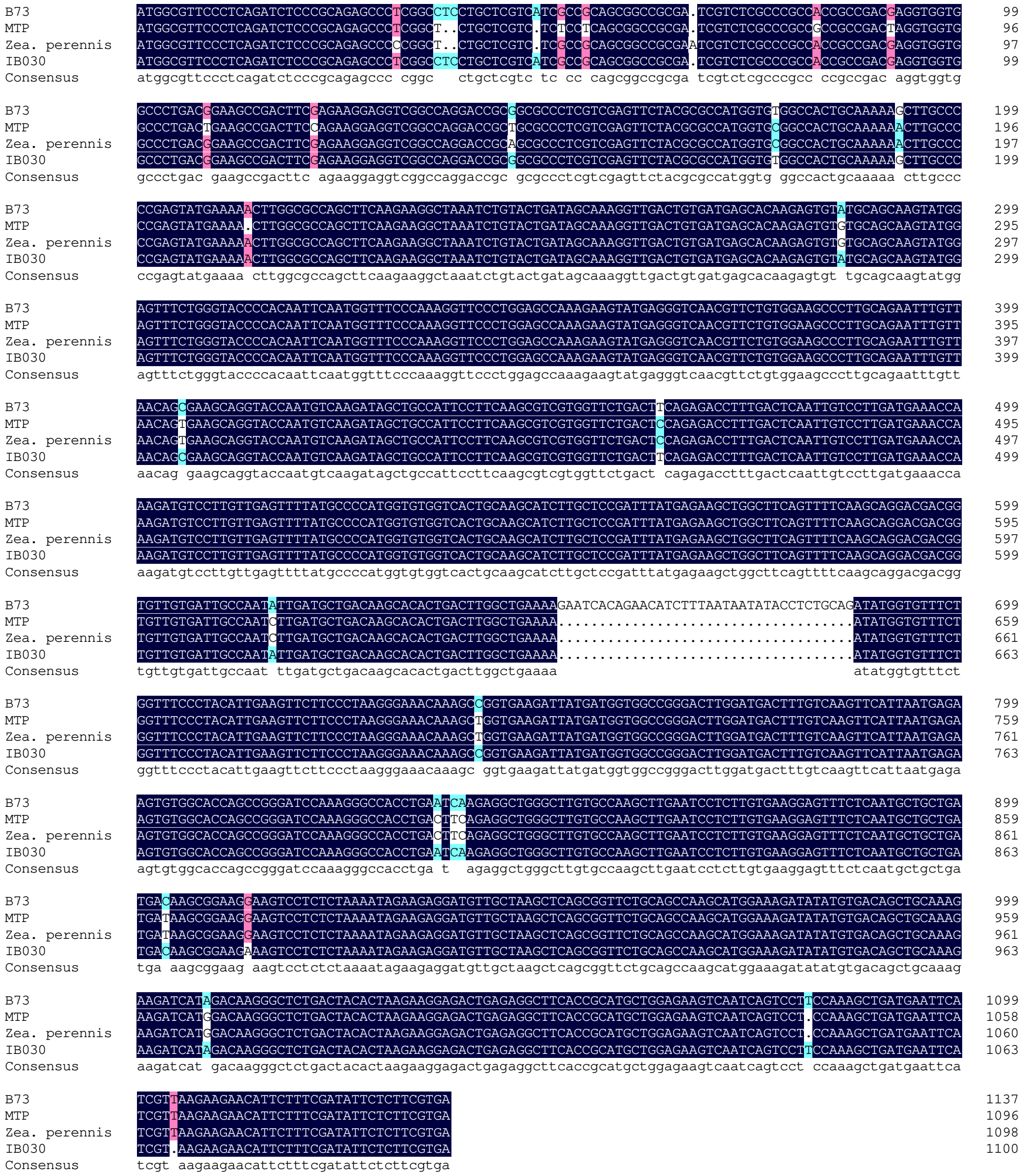

**Supplementary Figure S10**. Sequence comparison of homologous clones of *Zm00001d037590*

Note: MTP (M: maize; T: T. *dactyloides* and P: *Z. perennis*).
