# Supplementary material for: QTL Mapping and a Transcriptome Integrative Analysis Uncover the Candidate Genes That Control the Cold Tolerance of Maize Introgression Lines at the Seedling Stage
Source: Int J Mol Sci. 2023 Jan 30;24(3):2629. doi: 10.3390/ijms24032629 (PMC9917090; doi:10.3390/ijms24032629)
Supplement: Supplementary file 1 [file ijms-24-02629-s001.zip › Supplementary Paper S1-6.pdf]

Supplementary Paper S1 Cloning primers for candidate genes

Gene	Forward primer	Reverse primer
Zm00001d012321	CAGTCCACCATCGAACGACA	AAAAGGCCCCAACGAAATGC
Zm00001d037590	ATGGCGTTCCCTCAGATCTCC	TCACGAAGAGAATATCGAAAG

Supplementary Paper S2 Preparation of PCR reaction solution

Reagent	Usage amount/μl
PrimeSTAR Max Premix（2×）	25
Primer 1	1.5
Primer 2	1.5
Template	2
ddH_2O_2	20

Supplementary Paper S3 Components of qRT-PCR reaction

Reagent	Volume (μL)
ddH_2O_2	2
2X SYBR Green Fast qPCR Mix	5
Forward primer（10μM）	1
Reverse primer（10μM）	1
cDNA	1
Total	10

Supplementary Paper S4 Primers used in RT-PCR

Gene	Forward primer	Reverse primer
Zm00001d012321	TTATCGTTCTCGAGGCCAGC	GGCTCCGGTTGAACTCTCTC
Zm00001d037590	TTCTGCAGCCAAGCATGGAA	CCGTAAAGTGGTGGTTCGCT

Supplementary Paper S5 Primers of internal control genes used in RT-PCR

Gene	Forward primer	Reverse primer
ZMGAPDH	CCATCACTGCCACACAGAAAAC	AGGAACACGGAAGGACATACCAG
ZmACTIN	TCACCCTGTGCTGCTGACCG	GAACCGTGTGGCTCACACCA

Supplementary Paper S6 the determination of the M value according to the protocol described

1. Experimental design

1) Definition of experimental and control groups

Experimental groups: MIL-IB030 and B73 were treated at 2℃ for 0h, 2h, 6h, 12h and 24h at seedling stage, respectively

Control groups: MIL-IB030 and B73 were treated at 25℃ for 0h, 2h, 6h, 12h and 24h at seedling stage, respectively

2) Number within each group

Experimental groups: 15 samples

Control groups: 15 samples

2. Sample

1) Description

RNA from the leaves of MIL-IB030 and B73 at seedling stage were treated at 25℃ and 2℃ for 0h, 2h, 6h, 12h and 24h, respectively.

2) Micro-dissection or macro-dissection

Macro-dissection

3) Processing procedure

The leaves of MIL-IB030 and B73 were collected after treatment at 25℃ and 2℃ for 0h, 2h, 6h, 12h and 24h, respectively.

4) If frozen, how and how quickly?

The samples were frozen in liquid nitrogen for 15 min

5) If fixed, with what and how quickly?

No

6) Sample storage conditions and duration

The samples were stored in -80 ℃ ultra-low temperature refrigerator for 1h.

3. Nucleic acid extraction

1) Procedure and/or instrumentation

Total RNA was extracted from leaves of the seedling of MIL-IB030 and B73 after treating them at 25 °C and 2 °C for 0 h, 2 h, 6 h, 12 h, and 24 h using HiPure Plant RNA

Mini Kit (Magen Biotech Co., Ltd) following manual instruction. After confirming the concentration and quality RNA by NanoVue Plus nucleic acid protein analyzer (Eppendorf, BioPhotometer, China), the total RNA was reverse transcribed to cDNA by using RevertAid First Strand cDNA Synthesis Kit (TaKaRa, Dalian, China) following manual instruction.

2) Name of kit and details of any modifications and Details of DNase or RNase treatment

Table Remove genomic contamination system in RNA

Remove genomic contamination system in RNA	
Reagent	Volume (μL)
10X Reaction Buffer	1
with $MgCl_2$	1
total RNA	1
DNase I	1
Water, nuclease-free	7
Total	10

3) Contamination assessment (DNA or RNA) and Method/instrument

RNA concentration and OD_{260} /OD_{280} values were detected by NanoVue Plus nucleic acid protein analyzer, and RNA sample quality was tested.

4. Reverse transcription

1) Complete reaction conditions

SynthesisKit cDNA synthesiskit (TaKaRa) was used RevertAid First Strand cDNA SynthesisKit kit (Takara). Total RNA of samples was used as template for reverse-transcription into cDNA. Genomic contamination was removed from RNA at the same time. Add 1 μL of 50 mM EDTA and incubate at 65℃ for 10 min

2) Amount of RNA and reaction volume

Table Reverse transcription system

Reverse transcription system	
Reagent	Volume (μL)
5X Reaction Buffer	4
Oligo (dT) 18 primer	1
10 mM dNTP Mix	2

total RNA	1
RiboLock Rnase Inhibitor	1
RevertAid M-MuLV RT	1
Water, nuclease-free	10
Total	20

3) Priming oligonucleotide (if using GSP) and concentration

No

4) Reverse transcriptase and concentration

RiboLock Rnase Inhibitor

5) Temperature and time

The reverse transcription procedure was performed at 42℃ for 60 min. After 5 min at 72℃ and 12℃, the samples were stored at -20℃ for use.

5. qPCR target information

1) Gene symbol

Zm00001d012321 and *Zm00001d037590*

2) Sequence accession number

Zm00001d012321: LOC100382754

Zm00001d037590: LOC606414

3) Amplicon length

Zm00001d012321: 1425bp

Zm00001d037590: 4643bp

4) Location of each primer by exon or intron (if applicable)

Zm00001d012321: The first exon

Zm00001d037590: The first exon

6. qPCR oligonucleotides

1) Primer sequences

Gene	Forward primer	Reverse primer
Zm00001d012321	TTATCGTTCTCGAGGCCAGC	GGCTCCGGTTGAACTCTCTC
Zm00001d037590	TTCTGCAGCCAAGCATGGAA	CCGTAAAGTGGTGGTTCGCT

2）RT Primer DB identification number

No

7. qPCR protocol

1) Complete reaction conditions, Reaction volume and amount of cDNA/DNA, and Primer, probe, Mg^{2+}, and dNTP concentrations

Table RT-PCR system

Reagent	Volume (μL)
ddH_2O_2	5
2X SYBR Green Fast qPCR Mix	7.5
Forward primer（10μM）	0.4
Reverse primer（10μM）	0.4
cDNA	1
Total	10

4) Polymerase identity and concentration

2X SYBR Green Fast qPCR Mix

5) Buffer/kit identity and manufacturer

No

6) Complete thermocycling parameters

Table RT-PCR reaction procedure

Step	Temperature	Time
1	95 ℃	3 min
2	95 ℃	5s
3	60 ℃	30 s
4	+ H10Plate Read，Go to step 2	40-45 cycles
5	Melt Curve 65-95℃	
6	+ Plate Read，increment 0.5℃	0.5 s
7	end	

7）Manufacturer of qPCR instrument

The CFX96 real-time PCR system (Bio-Rad)

8. Data analysis

1) qPCR analysis program (source, version)

The real-time PCR data were analyzed by the comparative CT method (Schmittgen et al., 2008. doi:10.1038/nprot.2008.73).

2) Method of Cq determination

The Cq value is the number of PCR cycles at the intersection of the sample response curve and the threshold line. The PCR instrument software calculates the Cq value of each sample.

3) Justification of number and choice of reference genes

The *ZmACTIN* (*Zm00001d010159*) and *ZmGAPDH* (*Zm00001d049641*) genes were used as the internal control genes

4) Description of normalization method

Revised the accurate normalization of real-time quantitative RT-PCR data by geometric averaging of multiple internal control genes.

5) Number and stage (reverse transcription or qPCR) of technical replicates

Three biological replicates, four technical replicates.

6) Repeatability (intraassay variation)

There were four replicates within the group, and there was little variation.

7）Statistical methods for results significance

The real-time PCR data were analyzed by the comparative CT method, and revised the accurate normalization of real-time quantitative RT-PCR data by geometric averaging of multiple internal control genes.

8）Software (source, version)

Microsoft Excel (Redmond, WA, USA), SPSS (IBM, Inc., Armonk, NY, USA), and GraphPad Prism v9.0 (San Diego, CA, USA) were used for descriptive statistics.
