# Supplementary material for: QTL Mapping and a Transcriptome Integrative Analysis Uncover the Candidate Genes That Control the Cold Tolerance of Maize Introgression Lines at the Seedling Stage
Source: Int J Mol Sci. 2023 Jan 30;24(3):2629. doi: 10.3390/ijms24032629 (PMC9917090; doi:10.3390/ijms24032629)
Supplement: Supplementary file 1 [file ijms-24-02629-s001.zip › Supplementary Table S1-15.pdf]

Supplementary Table S1-I. Statistical analysis of phenotypic index of MTP- maize introgression line population（25℃；5 d）

Trait	Range	Mean	SE	CV/%	Kurtosis	Skewness	Sig
PH/cm	27.50-49.90	36.8604	0.6849	0.0186	-0.3308	0.3760	**
SFW/g	1.91-6.54	3.8109	0.1590	0.0417	0.1742	1.0088	**
SDW/g	0.19-0.72	0.4051	0.0190	0.0468	-0.2515	0.9044	**
RFW/g	1.30-4.77	2.8369	0.0928	0.0327	-0.1139	-0.0683	**
RDW/g	0.13-0.43	0.2939	0.0108	0.0367	-0.9612	0.1681	**
REC	0.08-0.60	0.2721	0.0166	0.0609	0.5613	1.0469	**

Note: ns is not significant; ** means significant at 0.01 level. Plant height/PH, seedling fresh weight/SFW, seedling dry weight/SDW, relative electrical conductivity/REC, root fresh weight/RFW and root dry weight/RDW.

Supplementary Table S1-II. Statistical analysis of phenotypic index of MTP- maize introgression line population（2℃；5 d）

Trait	Range	Mean	SE	CV/%	Kurtosis	Skewness	Sig
PH/cm	16.33-47.30	28.1188	1.0540	0.0375	-0.7627	0.6780	**
SFW/g	0.47-2.36	1.0466	0.0667	0.0637	-0.5402	0.7378	**
SDW/g	0.08-0.67	0.2717	0.0217	0.0799	-0.3232	0.8905	**
RFW/g	0.34-2.69	1.1299	0.0826	0.0731	-1.0566	0.4122	**
RDW/g	0.03-0.69	0.2084	0.0218	0.1048	1.1382	1.3621	**
REC	0.06-0.67	0.3697	0.0223	0.0603	-1.1149	0.4718	**

Note: ns is not significant; ** means significant at 0.01 level. Plant height/PH, seedling fresh weight/SFW, seedling dry weight/SDW, relative electrical conductivity/REC, root fresh weight/RFW and root dry weight/RDW.

Supplementary Table S2. Grade division of cold resistance of 21 maize introgression group materials

Order	Category	Material	Membership value
1	High cold tolerance type	IB030	0.7237
2	Cold resistance type	IZ089	0.5680
3		IB011	0.5662
4		IB010	0.5547
5		IB024	0.5233
6		IB018	0.5158
7		IB026	0.5128
8		IB015	0.5099
9		IZ054	0.5048
10	Cold sensitive type	Mo17	0.4395
11		IM001	0.4235
12		Chang7-2	0.3955
13		IB017	0.3622
14		IB025	0.3162
15		Zheng58	0.3066
16		IC099	0.3035
17	Alpine sensitive type	IM003	0.2374
18		IZ080	0.2239
19		IB023	0.1893
20		B73	0.1614
21		IB021	0.1368

Supplementary Table S3. Statistical analysis of parental phenotypic indicators in the plotting population (25℃; 5 d)

Trait	Range	Mean	SE	CV/%	Kurtosis	Skewness	Sig
PH/cm	28.62-46.90	38.6537	2.2776	0.0589	-1.3105	-0.4597	**
SFW/g	1.90-6.54	3.2730	0.4696	0.1435	3.6562	1.8783	ns
SDW/g	0.19-0.71	0.3513	0.0559	0.1591	1.7129	1.5497	*
RFW/g	1.30-3.72	2.4118	0.2909	0.1206	-1.2359	-0.2647	**
RDW/g	0.13-0.43	0.2630	0.0381	0.1448	-1.3196	0.3481	**
REC	0.08-0.28	0.1792	0.0214	0.1193	-0.2638	0.1942	*
RL/cm	400.61-530.44	466.3885	14.8303	0.0318	-1.3982	-0.1791	ns
RL/PH	9.00-17.10	12.4865	0.9792	0.0784	-0.8334	0.7054	*

Note: ns is not significant; ** means significant at 0.01 level. Plant height/PH, seedling fresh weight/SFW, seedling dry weight/SDW, root fresh weight/RFW, root dry weight/RDW, relative electrical conductivity/REC, root length/RL and RL/PH.

Supplementary Table S4. Statistical analysis of parental phenotypic indicators in the plotting population (2℃; 5 d)

Trait	Range	Mean	SE	CV/%	Kurtosis	Skewness	Sig
PH/cm	16.33-47.30	32.4870	3.9933	0.1229	-1.5653	-0.3500	**
SFW/g	0.52-1.93	1.0562	0.1668	0.1579	-0.8716	0.5726	**
SDW/g	0.09-0.40	0.1850	0.0393	0.2124	-0.6327	0.9968	**
RFW/g	0.45-1.57	1.0616	0.1585	0.1493	-1.8005	-0.4624	**
RDW/g	0.03-0.13	0.0833	0.0143	0.1718	-1.7657	-0.2803	*
REC	0.16-0.37	0.2801	0.0222	0.0793	-0.3499	-0.4592	*
RL/cm	118.90-429.48	228.2843	41.3803	0.1813	-1.2790	0.7860	**
RL/PH	4.02-9.48	6.9176	0.6366	0.0920	-0.7706	-0.1094	*

Note: ns is not significant; ** means significant at 0.01 level. Plant height/PH, seedling fresh weight/SFW, seedling dry weight/SDW, root fresh weight/RFW, root dry weight/RDW, relative electrical conductivity/REC, root length/RL and RL/PH.

Supplementary Table S5. Polymorphism screening of SSR markers between parents

SSR makers	Chr	SSR makers	Chr	SSR makers	Chr	SSR makers	Chr	SSR makers	Chr	SSR makers	Chr	SSR makers	Chr
bnlg1811	1	umc1798	2	umc1144	3	umc2287	4	umc1221	5	bnlg657	7	umc1570	9
bnlg2086	1	bnlg1141	2	umc1394	3	umc1940	4	umc1048	5	umc1944	7	umc1967	9
bnlg2295	1	phi328189	2	umc2277	3	umc1620	4	umc2373	5	umc1408	7	umc1522	9
umc2228	1	umc1542	2	umc2267	3	bnlg1137	4	umc2291	5	phi034	7	umc2370	9
bnlg1025	1	umc1755	2	bnlg2241	3	umc1232	4	umc1829	5	umc1694	7	umc1636	9
umc2240	1	umc2085	2	umc2050	3	umc1707	4	umc2304	5	umc1103	7	umc1675	9
bnlg1502	1	umc1518	2	umc1970	3	phi006	4	umc2296	5	umc2379	7	umc1893	9
bnlg1188	1	umc2363	2	umc1273	3	umc1869	4	umc2198	5	bnlg1666	7	bnlg1372	9
umc1630	1	mmc0491	2	bnlg1019	3	umc2082	4	umc2143	5	umc1913	8	bnlg1583	9
umc2233	1	bnlg1613	2	umc1813	3	umc2284	4	umc1656	6	umc1817	8	bnlg1272	9
umc1547	1	umc1448	2	umc1892	3	umc1643	4	umc1595	6	bnlg1823	8	umc1657	9
umc1306	1	umc1560	2	umc1773	3	umc2289	4	umc1918	6	umc1327	8	umc1366	9
umc2385	1	umc2079	2	umc2263	3	umc1067	4	umc1023	6	umc1807	8	umc1271	9
umc1797	1	umc1637	2	umc2118	3	bnlg1660	5	bnlg1617	6	bnlg1599	8	umc1794	9
umc2231	1	umc1541	2	umc2039	4	mmc0481	5	umc2170	6	umc1034	8	phi059	10
mmc0031	1	umc1259	2	umc1775	4	bnlg1885	5	dupssr15	6	bnlg1194	8	umc2021	10
umc1848	1	umc1756	2	umc1303	4	bnlg1847	5	bnlg1759	6	umc1470	8	bnlg1028	10
bnlg1671	1	umc2249	2	umc1821	4	umc1815	5	bnlg345	6	bnlg1152	8	umc1863	10
umc1353	1	bnlg1831	2	umc1142	4	bnlg1208	5	umc2375	6	umc1997	8	umc1336	10
umc1812	1	umc2246	2	bnlg1917	4	bnlg2323	5	phi123	6	umc1457	8	umc2043	10
umc1972	1	umc1422	2	bnlg1370	4	umc1482	5	umc2320	6	bnlg1031	8	phi118	10
phi227562	1	bnlg1909	2	umc1043	4	umc2293	5	umc2162	6	bnlg2181	8	dupssr8	10
umc1323	1	umc1138	2	umc1173	4	bnlg609	5	phi382202	6	umc1562	8	umc1319	10
umc1396	1	bnlg1329	2	bnlg1444	4	umc2013	5	umc2309	6	bnlg1460	8	phi052	10

bnlg1627	1	bnlg1940	2	phi072	4	bnlg2305	5	umc1018	6	umc1473	8	bnlg1451	10
umc1160	1	bnlg1520	2	umc1276	4	umc1226	5	umc1796	6	umc2075	8	umc2348	10
umc1493	1	umc1252	2	umc1759	4	bnlg1306	5	nc013	6	umc1858	8	umc2180	10
umc1601	1	umc1814	3	nc004	4	umc2136	5	bnlg2249	6	umc1263	8	bnlg1526	10
phi001	1	umc1501	3	umc1738	4	umc2038	5	umc2055	6	mmc0181	8	umc1115	10
umc1928	1	mmc0071	3	umc1610	4	umc1225	5	phi299852	6	umc1268	8	umc2221	10
phi037	1	bnlg1447	3	bnlg1189	4	umc2307	5	umc1127	6	bnlg1131	8	umc1176	10
umc2204	1	bnlg109	3	phi096	4	bnlg557	5	bnlg1136	6	umc2131	9	phi117	10
umc1605	1	bnlg1904	3	umc1669	4	bnlg118	5	umc1549	7	bnlg1091	9	umc1873	10
umc1552	2	umc1135	3	umc1871	4	phi085	5	bnlg1094	7	umc1957	9	umc1196	10
umc2248	2	umc2272	3	umc1086	4	phi048	5	bnlg1792	7	umc1191	9	umc1053	10
umc2247	2	umc2257	3	umc1667	4	mmc0081	5	umc1016	7	bnlg1209	9	umc1038	10
umc1769	2	umc1140	3	umc1791	4	bnlg1597	5	umc1213	7	umc1982	9	umc1993	10
bnlg381	2	umc1449	3	umc2286	4	bnlg389	5	umc1684	7	umc1137	9	umc1477	10
umc1165	2	umc2266	3	umc2365	4	umc1523	5	umc1987	7	umc2084	9	bnlg1360	10
umc2030	2	umc1320	3	umc1999	4	umc1679	5	umc1583	7	umc1094	9	dupssr30	10

Supplementary Table S6. Characteristics of linkage genetic map of $F_{2:3}$ populations

Number	Chromosome	Number of markers	Length(cM)	Average distance (cM /maker)
1	Chr1	33	924.70	28.03
2	Chr2	33	698.27	21.16
3	Chr3	27	718.89	27.54
4	Chr4	41	1005.11	26.63
5	Chr5	36	801.91	22.28
6	Chr6	19	535.44	28.18
7	Chr7	17	487.20	28.66
8	Chr8	25	556.04	22.24
9	Chr9	23	810.32	35.32
10	Chr10	26	909.41	34.98
Total		280	7449.51	26.60

Supplementary Table S7. QTL mapping results of $F_{2:3}$ population-related traits (2℃;5 d)

Trait	QTL	Chromosome	Marker interval	LOD	PVE	Additive effect
REC	*qREC2-1*	2	umc1798-bnlg1940	3.73	2.66	-0.0454
REC	*qREC3-1*	3	umc1892-umc2118	3.08	2.09	0.0383
REC	*qREC3-2*	3	umc1394-umc1144	5.22	3.75	-0.0497
REC	*qREC6-1*	6	bnlg2249-umc1918	4.82	3.46	0.0035
REC	*qREC8-1*	8	umc1268-bnlg1152	3.57	6.03	0.0046
PH	*qPH3-1*	3	umc1892-umc2118	5.65	8.48	-0.5848
PH	*qPH5-1*	5	umc2296-umc2143	3.25	6.62	-1.6670
SFW	*qSFW2-1*	2	bnlg1831-bnlg1909	3.20	1.88	0.0527
SFW	*qSFW3-1*	3	umc2118-umc1970	3.15	6.55	-0.0582
SFW	*qSFW4-1*	4	umc1620-umc1667	6.51	8.77	0.0304
SFW	*qSFW5-1*	5	umc2296-umc2143	3.17	2.81	-0.0709
SFW	*qSFW6-1*	6	dupssr15-bnlg1136	3.92	5.27	-0.0499
SDW	*qSDW1-1*	1	umc1547-bnlg1671	5.59	5.21	-0.0001
SDW	*qSDW1-2*	1	bnlg1671-umc1396	5.62	5.37	-0.0006
SDW	*qSDW1-3*	1	umc1396-umc1323	3.59	0.95	-0.0077
SDW	*qSDW2-1*	2	bnlg1329-umc1637	3.72	1.00	0.0055
SDW	*qSDW3-1*	3	umc2118-umc1970	4.57	3.39	-0.0126
SDW	*qSDW3-2*	3	mmc0071-umc1394	3.47	2.66	-0.0165
SDW	*qSDW3-3*	3	umc1394-umc1144	3.51	3.11	-0.0155
SDW	*qSDW3-4*	3	umc2263-umc2272	3.71	4.99	-0.0002
SDW	*qSDW4-1*	4	umc1276-umc1759	4.25	5.59	-0.0029
SDW	*qSDW4-2*	4	umc1620-umc1667	3.99	1.41	0.0076
SDW	*qSDW5-1*	5	umc2143-umc1221	5.79	1.59	-0.0114
SDW	*qSDW6-1*	6	bnlg1136-bnlg345	7.20	2.72	-0.0059

RL/PH	*qTRLRPH2-1*	2	umc1798-bnlg1940	7.41	10.57	0.6498

Note: Plant height/PH, seedling fresh weight/SFW, seedling dry weight/SDW, relative electrical conductivity/REC and root length/RL.

Supplementary Table S8. Summary of sample sequencing data quality

Sample	Raw_reads	Clean_reads	Q20	Q30	GC_pct
IB030_T1_72h	45293300	43319312	98.25	94.54	55.93
IB030_T2_72h	46598674	44421634	98.14	94.47	55.85
IB030_T3_72h	47257836	45060562	98.18	94.61	55.97
B73_T1_72h	46160272	44507132	98.17	94.58	56.27
B73_T2_72h	45431270	43876586	98.19	94.51	54.45
B73_T3_72h	47406382	44847570	98.13	94.31	55.71
IB030_CK1_72h	46424364	44134824	98.16	94.45	54.25
IB030_CK2_72h	47348880	44577004	98	93.94	53.59
IB030_CK3_72h	48957344	45673488	98.17	94.42	52.62
B73_CK1_72h	46784020	44081748	98.23	94.57	52.5
B73_CK2_72h	45441914	42236136	98.23	94.53	52.19
B73_CK3_72h	45520554	42554038	98.18	94.48	53.49
IB030_T1_120h	46289862	43978916	98.13	94.47	55.72
IB030_T2_120h	46642386	44209096	98.08	94.33	56.28
IB030_T3_120h	45330812	42837796	98.06	94.3	55.89
B73_T1_120h	47505738	45696638	98.05	94.22	55.81
B73_T2_120h	45285034	43326904	97.94	93.99	55.64
B73_T3_120h	45445498	43348244	98.03	94.17	55.87
IB030_CK1_120h	46544866	44655736	98.24	94.62	55.35
IB030_CK2_120h	43699626	41820028	98.27	94.73	54.26
IB030_CK3_120h	45813758	43560182	98.14	94.43	54.39
B73_CK1_120h	47285340	44490548	98.13	94.38	54.61
B73_CK2_120h	46110870	43611780	98.27	94.72	53.77
B73_CK3_120h	44011070	41539550	98.21	94.57	53.94

IB030_T1_HF24h	44020198	42085690	98.41	95.12	54.64
IB030_T2_HF24h	45976328	43788536	98.23	94.64	55.02
IB030_T3_HF24h	47640654	45223652	97.65	93.1	54.99
B73_T1_HF24h	45289206	43301894	97.85	93.77	54.51
B73_T2_HF24h	49112830	45117332	98.17	94.39	53.76
B73_T3_HF24h	45373514	42634346	98.02	94.09	53.76
IB030_CK1	46472838	44207410	98.28	94.74	55.27
IB030_CK2	45743312	43592920	98.21	94.58	54.86
IB030_CK3	43772726	41536690	98.15	94.41	54.65
B73_CK1	45981480	43253594	98.17	94.47	53.76
B73_CK2	46338758	43156456	98.11	94.32	53.15
B73_CK3	47372592	44483582	98.24	94.62	53.73

Supplementary Table S9. Sample and reference genome comparison statistics

Sample	Total_reads	Total_map	Proper_map
IB030_T1_72h	43319312	37775963(87.2%)	34398004(79.41%)
IB030_T2_72h	44421634	38360717(86.36%)	34835232(78.42%)
IB030_T3_72h	45060562	39238241(87.08%)	35807724(79.47%)
B73_T1_72h	44507132	42492752(95.47%)	40606456(91.24%)
B73_T2_72h	43876586	40967255(93.37%)	38773976(88.37%)
B73_T3_72h	44847570	43031457(95.95%)	40958922(91.33%)
IB030_CK1_72h	44134824	40240597(91.18%)	37086446(84.03%)
IB030_CK2_72h	44577004	40289989(90.38%)	37298236(83.67%)
IB030_CK3_72h	45673488	41023021(89.82%)	37917084(83.02%)
B73_CK1_72h	44081748	42803737(97.1%)	40677190(92.28%)
B73_CK2_72h	42236136	40926240(96.9%)	38887936(92.07%)
B73_CK3_72h	42554038	41201081(96.82%)	39024146(91.7%)
IB030_T1_120h	43978916	38493768(87.53%)	35044932(79.69%)
IB030_T2_120h	44209096	38419968(86.91%)	35063190(79.31%)
IB030_T3_120h	42837796	37165023(86.76%)	33728122(78.73%)
B73_T1_120h	45696638	42451446(92.9%)	39680868(86.84%)
B73_T2_120h	43326904	40519050(93.52%)	38446872(88.74%)
B73_T3_120h	43348244	39695683(91.57%)	37969358(87.59%)
IB030_CK1_120h	44655736	40446469(90.57%)	37309282(83.55%)
IB030_CK2_120h	41820028	37913479(90.66%)	34953444(83.58%)
IB030_CK3_120h	43560182	39600793(90.91%)	36533176(83.87%)
B73_CK1_120h	44490548	43298403(97.32%)	41103562(92.39%)
B73_CK2_120h	43611780	41989790(96.28%)	39839168(91.35%)
B73_CK3_120h	41539550	40160819(96.68%)	38131884(91.8%)

IB030_T1_HF24h	42085690	37702719(89.59%)	34765662(82.61%)
IB030_T2_HF24h	43788536	39665970(90.59%)	36651742(83.7%)
IB030_T3_HF24h	45223652	40617844(89.82%)	37496686(82.91%)
B73_T1_HF24h	43301894	41493553(95.82%)	39785724(91.88%)
B73_T2_HF24h	45117332	43354074(96.09%)	41630180(92.27%)
B73_T3_HF24h	42634346	41331764(96.94%)	39557336(92.78%)
IB030_CK1	44207410	40229089(91.0%)	37171498(84.08%)
IB030_CK2	43592920	39484979(90.58%)	36400182(83.5%)
IB030_CK3	41536690	37661074(90.67%)	34751128(83.66%)
B73_CK1	43253594	42065112(97.25%)	40041574(92.57%)
B73_CK2	43156456	41727409(96.69%)	39748482(92.1%)
B73_CK3	44483582	43366344(97.49%)	41237300(92.7%)

Supplementary Table S10. Details of hub genes detected by WGCNA

Gene	COG	KOG	Swissprot	NR
Zm00001d052201	-	Function unknown	Probable magnesium transporter NIPA8	hypothetical protein
Zm00001d052096	-	Lipid transport and metabolism	-	4-coumarate--CoA ligase-like 1
Zm00001d052097	-	Amino acid transport and metabolism	Probable gamma-aminobutyrate transaminase 3	hypothetical protein
Zm00001d012313	Carbohydrate transport and metabolism	-	-	unknown
Zm00001d037538	Translation, ribosomal structure and biogenesis	Translation, ribosomal structure and biogenesis	-	uncharacterized protein LOC100273441 isoform X2
Zm00001d037590	Posttranslational modification, protein turnover, chaperones	Posttranslational modification, protein turnover, chaperones	Protein disulfide isomerase	protein disulfide isomerase7 precursor
Zm00001d037605	-	Transcription	GATA transcription factor 23	GATA transcription factor 23-like

Zm00001d011971	Lipid transport and metabolism	Lipid transport and metabolism	1,4-dihydroxy-2-naphthoyl-CoA synthase, peroxisomal	delta3,5-delta2,4-dienoyl-CoA isomerase
Zm00001d012321	-	-	Nematode resistance protein-like HSPRO2	hypothetical protein ZEAMMB73_545725
Zm00001d037551	-	Signal transduction mechanisms	Probable GTP diphosphokinase CRSH1	hypothetical protein
Zm00001d011879	-	-	-	hypothetical protein
Zm00001d037602	-	RNA processing and modification	U1 small nuclear ribonucleoprotein	unknown

Supplementary Table S11. Homologous genes of key candidate genes

Gene	Arabidopsis thaliana (thale cress)	Japanese rice	Brassica napus (rape)
Zm00001d037590	AT2G47470	Os05g0156300	LOC106438331
Zm00001d012321	NA	Os01g0855600	NA

Supplementary Table S12. Results of exon and intron of key gene

Gene	No.	Exon / Intron	Start	End	Start Phase	End Phase	Length
		5' upstream sequence					
	1	Zm00001d037590_T002-E1	137,553,052	137,553,224	0	2	173
		Intron 1-2	137,553,225	137,554,734			1,510
	2	Zm00001d037590_T002-E2	137,554,735	137,554,819	2	0	85
		Intron 2-3	137,554,820	137,554,985			166
	3	Zm00001d037590_T002-E3	137,554,986	137,555,083	0	2	98
		Intron 3-4	137,555,084	137,555,163			80
	4	Zm00001d037590_T002-E4	137,555,164	137,555,219	2	1	56
		Intron 4-5	137,555,220	137,555,778			559
	5	Zm00001d037590_T002-E5	137,555,779	137,555,896	1	2	118
		Intron 5-6	137,555,897	137,555,990			94
Zm00001d037590	6	Zm00001d037590_T002-E6	137,555,991	137,556,018	2	0	28
		Intron 6-7	137,556,019	137,556,103			85
	7	Zm00001d037590_T002-E7	137,556,104	137,556,195	0	2	92
		Intron 7-8	137,556,196	137,556,309			114
	8	Zm00001d037590_T002-E8	137,556,310	137,556,320	2	1	11
		Intron 8-9	137,556,321	137,556,412			92
	9	Zm00001d037590_T002-E9	137,556,413	137,556,594	1	0	182
		Intron 9-10	137,556,595	137,556,681			87
	10	Zm00001d037590_T002-E10	137,556,682	137,556,806	0	2	125
		Intron 10-11	137,556,807	137,556,927			121
	11	Zm00001d037590_T002-E11	137,556,928	137,557,027	2	0	100
		Intron 11-12	137,557,028	137,557,114			87

	12	Zm00001d037590_T002-E12	137,557,115	137,557,183	0	0	69
		3' downstream sequence					
		5' upstream sequence					
Zm00001d012321	1	Zm00001d012321_T001-E1	173,684,485	173,685,910	0	0	1,425
		3' downstream sequence					

Supplementary Table S13. Cis-acting element of key candidate *Zm00001d012321*

Name	Sequences	Number	Function
CCAAT-box	CAACGG	1	MYBHv1 binding site
MYB recognition site	CCGTTG	1	MYB recognition site
TATA-box	TATAA	34	The element of the transcription initiation core promoter
AT-rich sequence	TAAAATACT	0	Maximum exciton mediates activation of the element
CAAT-box	CAAT	31	Common cis-acting elements in promoter and enhancer regions
ARE	AAACCA	3	Anaerobic induction homeopathic element
STRE	AGGGG	2	Stress response element
G-box	CACGAC	3	Cis-regulatory elements involved in light response
O2-site	GATGACATGG	1	Cis-regulatory element involved in regulation of zein metabolism
TGACG-motif	TGACG	2	Cis-acting regulatory elements involved in the response of methyl jasmonate
ABRE	CGTACGTGCA	3	Cis-acting elements involved in abscisic acid response
ERE	ATTTTAAA	2	Ethylene response element
CAT-box	GCCACT	1	Cis-acting regulatory elements associated with meristem expression

Supplementary Table S13. Cis-acting element of key candidate *Zm00001d037590*

Name	Sequences	Number	Function
CCAAT-box	CAACGG	0	MYBHv1 binding site
MYB recognition site	CCGTTG	0	MYB recognition site
TATA-box	TATAA	68	The element of the transcription initiation core promoter
AT-rich sequence	TAAAATACT	0	Maximum exciton mediates activation of the element
CAAT-box	CAAT	39	Common cis-acting elements in promoter and enhancer regions
ARE	AAACCA	2	Anaerobic induction homeopathic element
STRE	AGGGG	2	Stress response element
G-box	CACGAC	9	Cis-regulatory elements involved in light response
O2-site	GATGACATGG	1	Cis-regulatory element involved in regulation of zein metabolism
TGACG-motif	TGACG	2	Cis-acting regulatory elements involved in the response of methyl jasmonate
ABRE	CGTACGTGCA	7	Cis-acting elements involved in abscisic acid response
ERE	ATTTTAAA	1	Ethylene response element
CAT-box	GCCACT	0	Cis-acting regulatory elements associated with meristem expression

Supplementary Table S14. Identification of homologous Gene of *Zm00001d012321*

Variety	Query id	% identity	alignment length	mismatches	gap openings	q. Start	q. End	s. Start	s. End	E - value	score
B97_1	Zm00018ab384730	97.04	473	14	0	2	474	24	496	0	915
B97_2	Zm00018ab152070	86.221	479	50	7	1	474	1	468	0	789
CML52_1	Zm00019ab354630	93.939	495	9	1	1	474	1	495	0	914
CML52_2	Zm00019ab139410	86.221	479	50	7	1	474	1	468	0	790
CML247_1	Zm00023ab377980	96.617	473	15	1	2	474	24	495	0	908
CML247_2	Zm00023ab151410	86.013	479	51	7	1	474	1	468	0	786
DK105_1	Zm00016a039448	98.101	474	9	0	1	474	1	474	0	928
DK105_2	Zm00016a015441	86.221	479	50	7	1	474	1	468	0	789
Ki3-1	Zm00029ab382730	93.737	495	10	1	1	474	1	495	0	914
Ki3-2	Zm00029ab152700	85.924	476	53	8	1	474	1	464	0	775
Ki11_1	Zm00030ab369500	97.04	473	14	0	2	474	24	496	0	915
Ki11_2	Zm00030ab147250	86.221	479	50	7	1	474	1	468	0	790
Ky21_1	Zm00031ab381690	100	474	0	0	1	474	1	474	0	948
Ky21_2	Zm00031ab152690	85.595	479	49	9	1	474	1	464	0	774
Mo17_1	HSPRO2_0	97.04	473	14	0	2	474	24	496	0	915
Mo17_2	HSPRO2_1	86.43	479	49	7	1	474	1	468	0	792
Mo18_1	Zm00034ab391410	96.617	473	16	0	2	474	24	496	0	914
Mo18_2	Zm00034ab156920	86.221	479	50	7	1	474	1	468	0	790
Ms71_1	Zm00035ab380760	97.04	473	14	0	2	474	24	496	0	915
Ms71_2	Zm00035ab152010	86.013	479	51	7	1	474	1	468	0	786
NC350_1	Zm00036ab378140	98.101	474	9	0	1	474	1	474	0	928
NC350_2	Zm00036ab151400	86.892	473	52	6	1	471	1	465	0	789
Oh43_1	Zm00039ab375030	100	474	0	0	1	474	1	474	0	948

Oh43_2	Zm00039ab149040	86.221	479	50	7	1	474	1	468	0	790
P39_1	Zm00040ab390690	96.617	473	16	0	2	474	24	496	0	914
P39_2	Zm00040ab157560	86.221	479	50	7	1	474	1	468	0	789
W22_1	Zm00004b027883	96.829	473	15	0	2	474	24	496	0	915
W22_2	Zm00004b018424	86.221	479	50	7	1	474	1	468	0	789
Zx-PI566673_1	Zx00001a012656	86.221	479	50	7	1	474	1	468	0	788

Supplementary Table S15. Identification of homologous Gene of *Zm00001d037590*

Variety	Query id	% identity	alignment length	mismatches	gap openings	q. Start	q. End	s. Start	s. End	E value	score
B97	Zm00001d037590	94.03	134	8	0	22	155	223	356	4.31E-93	263
	Zm00001d037590	94.03	134	8	0	13	146	223	356	1.27E-92	261
	Zm00001d037590	95.522	134	6	0	22	155	223	356	1.21E-94	267
	Zm00001d037590	86.585	82	4	1	44	118	136	217	2.17E-45	139
	Zm00001d037590	85.484	124	6	1	1	112	201	324	1.24E-72	209
	Zm00001d037590	90.86	186	5	1	375	548	138	323	4.62E-115	340
	Zm00001d037590	87.407	135	5	1	5	127	187	321	5.00E-80	234
	Zm00001d037590	86.585	82	4	1	178	252	136	217	1.85E-45	144
	Zm00001d037590	95.522	134	6	0	13	146	223	356	2.55E-94	266
	Zm00001d037590	88.321	137	4	1	33	157	187	323	3.57E-83	238
	Zm00001d037590	87.33	221	9	2	185	386	136	356	6.12E-136	382
DK105	Zm00001d037590	95.522	134	5	1	11	143	223	356	9.90E-94	264
	Zm00001d037590	86.127	173	12	1	140	300	184	356	5.36E-103	296
	Zm00001d037590	89.873	79	1	1	1	72	139	217	1.50E-47	142
	Zm00001d037590	90.11	182	6	1	4	173	142	323	2.53E-117	329
Ki3	Zm00001d037590	96.269	134	5	0	11	144	223	356	5.79E-96	270
	Zm00001d037590	90.164	183	6	1	82	252	141	323	5.92E-118	334
	Zm00001d037590	89.247	186	8	1	415	588	138	323	6.30E-113	335
	Zm00001d037590	88.824	170	7	1	1	158	187	356	1.79E-107	299
	Zm00001d037590	88.608	79	9	0	1	79	278	356	3.31E-47	141

	Zm00001d037590	91.045	134	12	0	1	134	225	358	4.86E-89	251
	Zm00001d037590	93.75	128	8	0	4	131	229	356	1.22E-86	245
	Zm00001d037590	86.425	221	11	2	139	340	136	356	2.76E-135	378
Ky21	Zm00001d037590	91.045	134	12	0	22	155	223	356	2.61E-89	253
	Zm00001d037590	89.617	183	7	1	73	243	141	323	6.29E-117	331
	Zm00001d037590	90.805	87	8	0	5	91	137	223	3.03E-53	166
Mo18	Zm00001d037590	93.388	121	8	0	413	533	239	359	5.13E-76	233
	Zm00001d037590	85.78	218	12	2	177	375	141	358	4.21E-131	369
	Zm00001d037590	87.407	135	5	1	59	181	187	321	1.08E-79	234
	Zm00001d037590	95.556	45	2	0	165	209	280	324	3.02E-23	86.7
	Zm00001d037590	87.097	124	4	1	1	112	201	324	5.38E-74	213
Ms71	Zm00001d037590	90.805	87	8	0	5	91	137	223	3.24E-53	166
	Zm00001d037590	96.269	134	5	0	22	155	223	356	1.93E-95	270
	Zm00001d037590	88.321	137	4	1	33	157	187	323	3.57E-83	238
	Zm00001d037590	87.097	217	9	2	16	213	140	356	3.54E-134	370
	Zm00001d037590	96.269	134	5	0	22	155	223	356	1.93E-95	270
	Zm00001d037590	87.912	182	10	1	200	369	178	359	1.17E-111	319
	Zm00001d037590	89.706	136	14	0	22	157	223	358	1.29E-89	254
NC350	Zm00001d037590	89.617	183	7	1	28	198	142	324	2.01E-117	332
	Zm00001d037590	87.407	135	5	1	59	181	187	321	2.09E-79	234
	Zm00001d037590	89.928	139	13	1	22	159	223	361	1.71E-91	261
	Zm00001d037590	90.805	87	8	0	5	91	137	223	1.16E-52	167
Oh43	Zm00001d037590	95.902	122	5	0	68	189	230	351	9.53E-82	238

	Zm00001d037590	85.542	83	5	1	185	260	136	218	4.91E-46	145
	Zm00001d037590	88.889	90	10	0	5	94	137	226	1.84E-53	167
	Zm00001d037590	86.425	221	11	2	139	340	136	356	2.76E-135	378
	Zm00001d037590	88.235	153	6	1	1	141	204	356	2.57E-95	268
	Zm00001d037590	90.86	186	5	1	275	448	138	323	7.74E-117	340
	Zm00001d037590	89.617	183	7	1	73	243	141	323	6.29E-117	331
	Zm00001d037590	89.017	173	7	1	28	188	184	356	1.75E-110	308
P39	Zm00001d037590	87.05	139	6	1	1	127	187	325	7.23E-82	240
	Zm00001d037590	89.247	186	8	1	453	626	138	323	7.26E-111	332
	Zm00001d037590	96.269	134	5	0	22	155	223	356	1.93E-95	270
	Zm00001d037590	87.805	82	3	1	185	259	136	217	2.13E-46	147
	Zm00001d037590	86.585	82	4	1	139	213	136	217	4.85E-46	144
	Zm00001d037590	89.617	183	7	1	57	227	142	324	1.21E-118	331
	Zm00001d037590	94.118	136	8	0	13	148	223	358	1.62E-94	266
	Zm00001d037590	86.301	146	8	1	46	179	178	323	7.13E-87	255
W22	Zm00001d037590	91.549	142	12	0	13	154	223	364	6.45E-94	267
	Zm00001d037590	95.968	124	5	0	57	180	228	351	7.31E-84	242
	Zm00001d037590	87.586	145	18	0	180	324	212	356	2.24E-87	257
	Zm00001d037590	86.585	82	4	1	186	260	136	217	2.66E-45	144
	Zm00001d037590	94.03	134	8	0	13	146	223	356	2.06E-91	261
	Zm00001d037590	85.43	151	17	1	643	788	228	378	9.87E-85	262
Teo	Zm00001d037590	88.623	167	7	1	117	271	190	356	2.96E-102	293
	Zm00001d037590	87.946	224	12	2	44	255	136	356	6.41E-137	384

	Zm00001d037590	87.591	137	5	1	2	126	187	323	3.66E-81	238
	Zm00001d037590	97.368	38	1	0	2	39	282	319	3.37E-20	75.9
	Zm00001d037590	81.538	195	19	2	807	984	184	378	4.10E-101	310
	Zm00001d037590	77.836	379	71	2	1	367	1	378	0	578
	Zm00001d037590	63.158	19	6	1	1	18	261	279	5.66E-04	25.4
B73	Zm00001d037590	87.075	147	7	1	54	188	178	324	1.04E-91	261
	Zm00001d037590	86.425	221	11	2	139	340	136	356	6.10E-135	377
	Zm00001d037590	86.425	221	11	2	139	340	136	356	2.87E-134	375
	Zm00001d037590	63.158	19	6	1	1	18	261	279	5.66E-04	25.4
Mo17	Zm00001d037590	64.286	14	5	0	9	22	193	206	2.68E-04	23.1
	Zm00001d037590	94.118	136	8	0	13	148	223	358	1.62E-94	266
	Zm00019ab270710	96.825	378	0	1	1	378	1	366	0	735
	Zm00019ab126200	77.311	357	68	2	1	356	1	345	0	541
	Zm00019ab353430	88.839	224	13	1	136	359	44	255	5.90E-138	393
	Zm00019ab375470	86.425	221	11	2	136	356	139	340	4.93E-131	379
	Zm00019ab222530	79.661	177	18	2	185	361	63	221	7.36E-91	273
CML52	Zm00019ab047310	78.698	169	24	1	133	301	18	174	2.68E-90	269
	Zm00019ab169740	79.394	165	22	1	137	301	5	157	9.61E-89	265
	Zm00019ab428170	78.182	165	24	1	137	301	5	157	3.08E-88	263
	Zm00019ab342090	87.591	137	5	1	187	323	33	157	6.57E-78	238
	Zm00019ab171900	77.143	105	15	1	137	232	5	109	2.40E-49	167
	Zm00019ab435040	95.556	45	2	0	280	324	102	146	1.85E-18	86.3
Ki11	Zm00030ab283250	96.825	378	0	1	1	378	1	366	0	735

	Zm00030ab131710	77.311	357	68	2	1	356	1	345	0	541
	Zm00030ab316940	87.33	221	9	2	136	356	185	386	3.05E-131	381
	Zm00030ab048490	88.542	192	10	1	133	324	18	197	1.77E-118	342
	Zm00030ab127970	90.323	186	6	1	138	323	381	554	7.60E-111	338
	Zm00030ab142710	81.098	164	19	1	138	301	1	152	2.42E-90	268
	Zm00030ab180180	79.394	165	22	1	137	301	5	157	9.46E-89	265
	Zm00030ab027840	94.03	134	8	0	223	356	22	155	1.82E-88	264
	Zm00030ab308930	95.349	129	6	0	228	356	112	240	1.02E-83	255
	Zm00030ab259880	87.857	140	5	1	184	323	35	162	3.28E-80	246
	Zm00030ab356980	87.591	137	5	1	187	323	33	157	5.10E-78	238
	Zm00030ab139860	87.407	135	5	1	187	321	59	181	9.36E-75	234
	Zm00030ab423630	85.401	137	8	1	187	323	72	196	9.15E-73	229
CML247	Zm00023ab291310	96.825	378	0	1	1	378	1	366	0	735
	Zm00023ab134910	77.311	357	68	2	1	356	1	345	0	541
	Zm00023ab376880	88.789	223	13	1	137	359	5	215	1.33E-138	393
	Zm00023ab153610	86.878	221	10	2	139	359	204	405	3.70E-129	376
	Zm00023ab184790	89.785	186	7	1	138	323	420	593	6.74E-110	339
	Zm00023ab430940	84.672	137	9	1	223	359	7	131	8.38E-76	230
	Zm00023ab322400	85.542	83	5	1	136	218	186	261	4.88E-41	145
	Zm00023ab430910	86.585	82	4	1	136	217	178	252	1.50E-40	144
